# Oligodeoxynucleotides Can Transiently Up- and Downregulate CHS Gene Expression in Flax by Changing DNA Methylation in a Sequence-Specific Manner

**DOI:** 10.3389/fpls.2017.00755

**Published:** 2017-05-15

**Authors:** Magdalena Dzialo, Jan Szopa, Tadeusz Czuj, Magdalena Zuk

**Affiliations:** ^1^Department of Genetic Biochemistry, Faculty of Biotechnology, University of WrocławWroclaw, Poland; ^2^Linum FoundationWroclaw, Poland; ^3^Department of Genetics, Plant Breeding and Seed Production, Wroclaw University of Environmental and Life SciencesWroclaw, Poland

**Keywords:** chalcone synthase, genetic engineering, ODNs, epigenetics, methylation

## Abstract

Chalcone synthase (CHS) has been recognized as an essential enzyme in the phenylpropanoid biosynthesis pathway. Apart from the leading role in the production of phenolic compounds with many valuable biological activities beneficial to biomedicine, CHS is well appreciated in science. Genetic engineering greatly facilitates expanding knowledge on the function and genetics of CHS in plants. The CHS gene is one of the most intensively studied genes in flax. In our study, we investigated engineering of the CHS gene through genetic and epigenetic approaches. Considering the numerous restrictions concerning the application of genetically modified (GM) crops, the main purpose of this research was optimization of the plant's modulation via epigenetics. In our study, plants modified through two methods were compared: a widely popular agrotransformation and a relatively recent oligodeoxynucleotide (ODN) strategy. It was recently highlighted that the ODN technique can be a rapid and time-serving antecedent in quick analysis of gene function before taking vector-mediated transformation. In order to understand the molecular background of epigenetic variation in more detail and evaluate the use of ODNs as a tool for predictable and stable gene engineering, we concentrated on the integration of gene expression and gene-body methylation. The treatment of flax with a series of short oligonucleotides homologous to a different part of CHS gene isoforms revealed that those directed to regulatory gene regions (5′- and 3′-UTR) activated gene expression, directed to non-coding region (introns) caused gen activity reduction, while those homologous to a coding region may have a variable influence on its activity. Gene expression changes were accompanied by changes in its methylation status. However, only certain (CCGG) motifs along the gene sequence were affected. The analyzed DNA motifs of the CHS flax gene are more accessible for methylation when located within a CpG island. The methylation motifs also led to rearrangement of the nucleosome location. The obtained results suggest high specificity of ODN action and establish a potential valuable alternative for improvement of crops.

## Introduction

For the last two decades, epigenetics has been considered as an equally important source of organism variability as genetic modifications. Epigenetic changes refer to the chromatin modifications that do not occur due to the interruption of the nucleic acid sequence, such as DNA methylation or various histone modifications (including methylation, acetylation, phosphorylation, and ubiquitination; Handy et al., 2011).

The adaptation of plants to variable environmental conditions is the result of evolutionarily developed genetic and epigenetic regulatory systems (Liu et al., 2015). In plants stress factors induce the development of epigenetic marks and thus leave a “trace” in a memory of plant resistance, which enables them to respond rapidly to the repeated stress condition (Iglesias and Cerdán, 2016). In order to modulate a particular feature in plants, direct methods are used. The most popular method of the targeted modification of plants is *Agrobacterium*-mediated genetic transformation, an effective method of introducing an exogenous gene into the plant genome. Unfortunately, the cultivation of genetically modified (GM) crops comes under strict legal regulations in many European countries. For this reason, alternatives to genetic transformation methods are highly desirable.

According to plant molecular biology, in the last few years great attention has been given to the short oligodeoxynucleotides (ODNs). Short oligonucleotide sequences are naturally present in the cells of prokaryotic and eukaryotic organisms, where they serve mostly regulatory functions. In the ODN strategy mostly sequences in the length range of 12–25 nt, homologous to the particular gene region in the plant genome, that are able to hybridize with the target sequence are used. The effect of the short oligonucleotides activity depends on the secondary structure, nucleotide composition and orientation of the sequence (Sun et al., 2005; Dinç et al., 2011). The strategy of introducing short ODNs into mammalian cells proved to be an effective method for modification and gene function studies (Sun et al., 2008). The lack of interference in the DNA sequence has prompted attempts to use the method of oligodeoxynucleotides as a tool for modifying plants. An important advantage of this method for plants is the ability to study the function of key genes and reduction of the pleiotropic effects that are a common problem encountered when creating genetically modified organisms.

The mechanism of ODN action is not fully understood yet. However, the crucial regulatory processes presumably involve modulation of the gene expression as an effect of siRNA (small interfering RNA) and DNA methylation (Wojtasik et al., 2014; Ci et al., 2015). The introduction of short oligodeoxynucleotides into the cell leads to hybridization of ODN to the homologous region in the transcript sequence. This event activates RNA-dependent RNA polymerase (RdRP), which synthesizes the complementary RNA strand and leads to the formation of double stranded RNA (dsRNA), which might be responsible for the gene repression, as an effect of RNA interference (RNAi) (Baulcombe, 2004). Short exogenous oligonucleotides can also activate particular genes in the process called RNA activation (RNAa). However, this occurs as long as ODNs are homologous with the regulatory sequences. In plants, it seems that the mechanism tends to be based on DNA methylation, whereas in mammalian cells it is associated with histone modification (Shibuya et al., 2009; Huang et al., 2010). DNA methylation is considered to be one of the most important epigenetic marks in plants. The process of induction or deprivation of a methyl residue is based on the recruitment of the methyltransferases by RdRP or polymerase IV and polymerase V complex (López et al., 2011). The level of methylation of particular regulatory or coding regions of genes may be regulated by the homologous ODN. Eventually, this event may lead to modulation of the gene expression level (Chinnusamy and Zhu, 2009).

The strategy of oligodeoxynucleotides, unlike short hairpin RNA (shRNA) or artificial microRNA technology, does not require insertion of the sequence into a plasmid before it is delivered to the cell. Short oligonucleotides are simple to use, due to the direct introduction into cells. Similarly, the sequences can be introduced as siRNA; however, in comparison to ODNs their design and synthesis are more complex and do not allow even a single mismatched nucleotide in a sequence, which may drastically reduce their efficacy. Moreover, in the case of siRNA, many reports have noted their action outside the gene sequence of interest (Dinç et al., 2011).

There are several ways to provide ODNs to plant cells in a tissue culture: treatment of pollen tubes (Liao et al., 2013), oligonucleotide transfer to cells by spraying, infiltration under vacuum (Wojtasik et al., 2014; Xie et al., 2014) or through forced osmosis prior to sucrose starvation (Sun et al., 2005, 2008). Although the first reports on delivery of short ODNs into plant cells appeared over 20 years ago (Tsutsumi et al., 1992), the potential of this technology has not yet been fully exploited. The fact is that the development of every new strategy requires detailed optimization. In the ODN strategy usually the experimental conditions are personalized for a particular plant species. The antisense ODNs were successfully introduced into the green tissue of barley for the first time by a research group of Sun et al. (Sun et al., 2005). The aim of the experiment was to analyze the function of the gene encoding the transcription factor SUSIBA2 by silencing the gene activity. Later, a more detailed study of starch synthesis was carried out in the genes encoding the SBEs by silencing the activity of their genes. Two antisense oligonucleotide sequences, SBEI and SBEIIA, resulted in a significant decrease in the activity of SBE (Sun et al., 2008).

Recently, the ODN method was also performed on flax, *Linum usitatissimum* L., where treatment with short oligonucleotides led to the induction of changes in the endogenous β-1,3-glucanase gene. The obtained plants presented overexpression of the studied gene and reduction in the genomic methylation level. Moreover, these plants exhibited enhanced resistance to the pathogenic fungus of flax *Fusarium*. This study confirmed the use of ODNs as an effective alternative method for vector-mediated transformation (Wojtasik et al., 2014).

Although flax is not recognized as a model plant, it draws attention due to its favorable properties that can be used widely in medicine, nutrition, cosmetology, and high-tech industry. For research purposes in the flax the parameters of the oil, fiber and the broad spectrum of secondary metabolites are intensively investigated. The secondary metabolites in flax are predominantly phenylpropanoids (such as flavonoids, lignans, and coumarins) and terpenoids.

**Figure 1:**
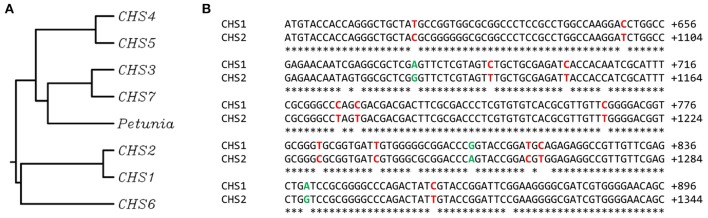
**Similarity between chalcone synthase gene sequences from flax ***Linum usitatissimum*** L. and ***CHS***-A from ***Petunia*** x ***hybrida***. (A)** The phylogenetic tree presents the similarity between following chalcone synthase gene sequences: *CHS*-A from *Petunia* x *hybrida* (GenBank: X04080), true *CHS* sequences from flax *CHS1* (Phytozome: Lus10031622) and *CHS2* (Phytozome: Lus10033717), and predicted *CHS* sequences from flax that display great resemblance to *CHS* sequences from other species: *CHS3* to *E. grandis* (GenBank: XM_010030619.2), *CHS4* to *E. guineensis* (GenBank: XM_010944042.1), *CHS5* to *R. graveolens* (GeneBank AJ297791.2), *CHS6* to *P. cuspidatum* (GenBank: EU647246.1) and *CHS7* to *H. lupulus* L. (GenBank: AM263200.1). **(B)** The alignment of the representative part of cDNA sequences of *CHS1* and *CHS2*. The substitutions between single nucleotides are marked (C/T in red, A/G in green).

Chalcone synthase (CHS) is considered to be one of the key enzymes in biosynthesis of flavonoids. In different species the amino acid sequences of CHS present a high level of similarity. However, the number of gene copies may differ greatly between the species: from a single one to multiple isoforms. For example, a single copy of the CHS gene was found in the model plant *Arabidopsis thaliana*. The majority of plant cultivars possess two copies of the CHS gene (*Oryza sativa, Zea mays, Hordeum vulgare*). In sugar cane (*Saccharum officinarum*) and *Petunia hybrida* at least 10 putative copies of the chalcone synthase gene were determined (Dao et al., 2011). In the genome of flax (*Linum usitatissimum* L.) 7 isoforms of the chalcone synthase gene were recognized (Figure 1A). Although a few of them are predicted to be true CHS sequences, *CHS1* and *2* are fully confirmed (Phytozome database, *CHS1* – Lus10031622, *CHS2* – Lus10033717). The similarity between coding sequences of isoforms 1 and 2 reaches 97%. The substitutions between sequences involve mostly the exchange between cytosine and thymine or adenine and guanine (Figure 1B). The mechanism of C to T transition is an effect of the spontaneous deamination of the 5-methyl cytosine (Cooper et al., 2010). The deamination process is also involved in the A to G transition, which leads to the formation of hypoxanthine, which is selectively paired to cytosine. Finally, complementary sequence synthesis results in the replacement of adenine with guanine (Talhaoui et al., 2014). These changes are considered also as epigenetic modifications, since there is no interruption of nucleic acid involved during the exchange process. In comparison to other organisms, these two isoforms share 67% similarity of the mRNA sequence with the chalcone synthase from *Petunia* x *hybrida* (GenBank acc. no. X04080). Other sequences display great resemblance to *CHS* sequences from other species: *CHS3* to *Eucalyptus grandis* (GenBank acc. no. XM_010030619.2), *CHS4* to *Elaeis guineensis* (GenBank acc. no. XM_010944042.1), *CHS5* to *Ruta graveolens* (GenBank acc. no. AJ297791.2), *CHS6* to *Polygonum cuspidatum* (GenBank acc. no. EU647246.1), and *CHS7* to *Humulus lupulus* L. (GenBank acc. no. AM263200.1).

Recently, the genetic transformation of flax by the introduction of the cDNA sequence of the CHS gene from *Petunia* x *hybrida* via *Agrobacterium tumefaciens* was performed. The new lines of transgenic plants showed the occurrence of both suppression and repression of the endogenous chalcone synthase gene. However, the application of genetically modified flax encounters numerous legal restrictions. Thus, the purpose of our research was the modulation of CHS gene expression due to induction of epigenetic changes in the pattern of the changes obtained through genetic transformation. In the study, in order to optimize the new strategy of plant modification, plants developed through two methods were compared: the widely popular agrotransformation and the relatively recent strategy of treatment with ODNs. The epigenetic modifications were induced in flax by short oligonucleotides homologous to particular regions of the endogenous chalcone synthase gene. The ODN sequences were introduced into the plant cells by vacuum infiltration. The expression of the endogenous *CHS* and methylation profiles were analyzed in the acquired flax lines. The effect of induction of epigenetic marks was compared to the flax developed due to genetic transformation. The stability of the genetic and epigenetic modulation was monitored at various points in time after treatment.

## Materials and methods

### Designing short oligonucleotide sequences (ODNS) homologous to CHS gene regions

Introns and exons in sequences were identified using the online tool GeneMark (http://exon.gatech.edu/GeneMark/). ODNs homologous to the various *CHS* regions were designed using Mfold software (Genetics Computer Group, USA). The software predicts the best sites of binding to the RNA sequence. In order to obtain the most active ODNs the nucleotide composition (%GC), secondary structure and melting temperature of the short sequences were taken into consideration. The 11 ODN sequences were designed in the antisense orientation for the CHS gene (Phytozome database, *CHS1* – Lus10031622, *CHS2* – Lus10033717). The short oligonucleotides were synthesized by Genomed S.A. (Poland). The parameters of the designed ODNs are presented in the Supplementary Table 1.

### Plant material

Flax seeds (*Linum usitatissimum* L., cv. *Linola*) were obtained from the Flax and Hemp Collection of the Institute of Natural Fibers (Poland). The seeds were germinated on Murashige and Skoog basal medium (Sigma-Aldrich), supplemented with 2% sucrose, pH 5.8, solidified with 0.8% agar on Petri dishes. Mature flax plants were cultured on Murashige and Skoog basal medium (MS medium), supplemented with 2% sucrose, pH 5.8, solidified with 0.4% agar and 0.1% gerlite in sterile glass jars. In order to minimize plant infection by pathogens, the medium was complemented with 0.1% PPM (Plant Cell Technology).

The plants were cultured in phytotron conditions of 16 h light (22°C), 8 h darkness (16°C).

### Transgenic plant generation and selection

#### Transformation of linola flax

Ten-day-old hypocotyls explants were transformed using *Agrobacterium tumefaciens* strain C58C1:pGV2260 carrying a binary vector containing cDNA encoding the chalcone synthase gene from *Petunia* x *hybrida* (*CHS*, EMBL/GenBank database accession no. X04080), in the sense orientation under the control of the 35S promoter and the OCS terminator. After 2 days of incubation with *Agrobacterium* inoculum, the plant material was rinsed in sterile water with antibiotics (Augmentin, Timentin) and transferred onto Petri plates with callus-inducing medium (CIM), which is the basal MS medium (supplemented with 2.5% sucrose, pH 5.8, solidified with 0.8% agar) with the suitable concentration of plant hormones stimulating callus formation (NAA 0.05 mg/L, BAP 1 mg/L). After 2 weeks the explants were transferred onto shoot-inducing medium (SIM)—basal MS medium (supplemented with 2.5% sucrose, pH 5.8, solidified with 0.8% agar) with addition of plant hormones, which helps in shoot formation (NAA 0.001 mg/L, BAP 0.02 mg/L). The newly developed shoots were cut and put into the sterile jars with MS medium solidified with agar.

#### Transgenic plant selection

The transgenic plants were selected by PCR using primers specific to the differences between nucleotide sequences of synthase chalcone genes from flax and petunia. Primers used in the reaction (amplified product—395 bp): forward—5′ TTTGGTCTTTTGCACAACTAGT 3′ (length = 22 nt, GC = 36%, Tm = 49.2°C); reverse—5′ AGATGGCCATCAATAGCACC 3′ (length = 20 nt, GC = 50%, Tm = 51.8°C). The actin gene was used as the reference. The introduction of the transgene was determined in the genomic DNA, which was isolated using the chloroform/phenol method. The reaction was carried out using Phusion polymerase (Thermo Scientific). Reaction conditions were designed following the manufacturer's protocol.

The amplified products were separated in 1.5% agarose gel. The DNA was stained by ethidium bromide and visualized in UV light (VisionWorks Image software).

#### Transgene expression analysis

Expression of the CHS transgene was analyzed via semi-quantitative PCR. The total RNA was isolated from freeze-ground green tissue of the transgenic plants. Isolation was performed using the Trizol method (Invitrogen), following the protocol of the manufacturer. The isolated RNA was deprived of DNA contamination by DNase I (Invitrogen). The purified RNA was used as a template for cDNA synthesis using reverse transcriptase—High Capacity cDNA Reverse Transcription Kit (Applied Biosystems).

Primers used in the reaction (amplified product: 539 bp): forward—5′ AGGCAAGACATAGTGGT 3′, reverse—5′ CCAGGAACATCTTTGAG 3′. The used reference gene was actin. The obtained cDNA was used as a template for the PCR with the Phusion polymerase. The reaction was performed in a different number of amplification cycles in order to determine the differences in transgene expression between bands. The PCR products were separated by gel electrophoresis (2% agarose, 0.2 μg/ml EtBr). The products were stained with ethidium bromide and visualized in UV light (VisionWorks Image software). HyperLadder (Bioline) 1 kb was used as the molecular weight marker.

### Flax treatment with ODNs

Four-week-old *Linola* flax cultured in *in vitro* conditions was used for the ODN treatment. The plants were cut above their roots and submerged in water solution of the particular ODN in 10 μM concentration. Incubation was performed in a vacuum chamber for 20 min. After the treatment the plants were put into Murashige-Skoog medium. The material for the analysis was harvested at various time points: 24 h, 48 h, and 10 days. The harvesting time was indicated by previous experiments and implied the occurrence of the most visible changes in plants after the ODN treatment in comparison to the control.

### Gene expression analysis

The expression of investigated genes was analyzed via real-time PCR. The total RNA was isolated using an identical method as in the transgene expression analysis, and it was used as a template for cDNA synthesis.

Real-time PCR reactions were performed using the DyNAmo SYBR Green qPCR kit (Thermo Scientific). Primers used for reactions are presented in the Supplementary Table 2. The used system was Applied Biosystems StepOnePlus Real Time PCR System. Reactions were performed according to the protocol of the manufacturer. Each reaction was performed in triplicate. The actin gene was used as the reference. The results were presented as the gene expression level relative to the reference gene.

### Total DNA methylation analysis

Genomic DNA from the analyzed plants was isolated using the DNeasy Plant Mini Kit (Qiagen) according to the manufacturer's protocol. For the analysis of ODN-treated plants the material was used 48 h after exposure.

The total methylation of the genomic DNA was assessed via the MethylFlash Methylated DNA Quantification Kit (Epigentek). The main principle of the kit is a colorimetric reaction of 5-methylcytosines in the analyzed DNA. The results were measured on 96-well microplates in the Varioscan Flash spectral reader (Thermo Scientific).

### DNA methylation patterns in specific regions of chalcone synthase gene

The methylation patterns of the chalcone synthase gene sequence were established in the control, transgenic and ODN-treated plants. For analysis of the ODN-treated plants the material was used 48 h after exposure. The DNA was incubated with restriction enzymes MspI and HpaII for at least 3 h (restriction enzymes MspI and HpaII (New England Biolabs) differ in sensitivity to cytosine methylation). The genomic DNA digested by the restriction enzymes and undigested DNA were used as templates for the real-time PCR reaction. The reaction was performed similarly to the gene expression analysis. The primers for the reaction (Supplementary Table 2) were designed for specific sites of methylation predicted by the NEB cutter V2.0. In the synthase chalcone gene six CCGG islands were analyzed. The sites of analyzed -CCGG- motifs were indicated by their positions toward the ATG site (+1) as follows: 5′-UTR (site −232), non-coding region (+217) and coding region (+996, +1,219, +1,273, +1,606).

The quantity of DNA measured by real-time PCR was calculated in order to estimate the methylation of cytosines. The “CCGG” non-methylated DNA was calculated as the difference between undigested DNA and DNA incubated with HpaII. The single methylated cytosine “CCmGG” was estimated as the difference between samples digested by HpaII and digested by MspI. The level of the “CmCmGG” was equal to the DNA incubated with MspI. The values were presented as percentages in reference to the undigested DNA, set as 100%.

### *In silico* study

#### Alignment of -CCGG- motifs in context of CHS gene

For the analysis, the nearest vicinity of 22 base pairs upstream and downstream of each *CHS1* and *CHS2* isoform was taken. In order to check the similarity between stable demethylated and variable sites selected sequences were aligned in terms of -CCGG- motifs and all nucleotides in each column were counted. These sequences also were evaluated for GC content (%) (Genomics %G ~ C Content Calculator), and whether they are located within a CpG island (http://www.ebi.ac.uk/Tools/seqstats/emboss_cpgplot/).

#### Energy landscape and nucleosome footprint

To detect possible nucleosome binding regions within a gene sequence, the *CHS* sequences were analyzed using the Interactive Chromatin Modeling tool (http://dna.engr.latech.edu/icm-du/) (Stolz and Bishop, 2010). As the results, the possible nucleosome footprint within the sequence and energy landscape (kcal/mol nucleotide) was obtained. Both sequences with methylated and unmethylated -CCGG- motifs (+996, +1,219, +1,273) were subjected to the nucleosome footprint analysis. These data were then confronted on charts, in order to visualize possible changes in the calculated energy landscape and nucleosome footprint.

### DNA accessibility for restriction

The DNA accessibility for restriction was established in the control and W.92 transgenic plants with stabilized modulation of CHS gene expression (Lorenc-Kukula et al., 2005) by using restriction enzymes AatII, PvuI, SacII, and Sau3AI (New England Biolabs). The first three enzymes were selected on the basis of the predicted cut sites in the vicinity of the +1,273 -CCGG- motif (NEB cutter V2.0). Sau3AI was selected due to its ability to digest the DNA at frequently occurring GATC sites. The genomic DNA was isolated using the DNeasy Plant Mini Kit (Qiagen) according to the manufacturer's protocol. The DNA was digested by particular enzymes for 1 h in the appropriate conditions (temperature, buffer) according to the attached protocols. The genomic DNA digested by the restriction enzymes and undigested DNA were used as templates for the real-time PCR reaction. For the analysis the +1,273 -CCGG- motif was selected on the basis of the methylation pattern experiment. The values were presented as relative quantity (RQ) in reference to the *Linola* control flax, set as 1.

### Statistical analysis

Presented experiments were performed at least three times, independently (three technical repeats of each of three biological samples). Data constitute the mean value ± standard deviation (SD) from at least three independent experiments. The significance of the differences between each mean and control was determined by Student's *t*-test. An asterisk above each bar indicates *p* < 0.05. The statistical analysis was performed using STATISTICA ver. 12 (StatSoft Inc., 2014).

## Results

### Generation and preliminary analysis of genetically modified flax (GM-CHS flax)

#### Selection of transgenic plants

The tool for the selection of transgenic lines was polymerase chain reaction (PCR). Due to 67% identity of CHS genes in flax (Phytozome database, *CHS1* – Lus10031622, *CHS2* – Lus10033717) and petunia (GenBank acc. no. X04080), it was crucial to design specific primers (see Materials and Methods section), by using slight differences between the nucleotide sequences of those heterologous CHS genes. Through the PCR reaction there were selected 8 lines, which presented introduction of the CHS transgene into the genomic DNA.

#### Expression of the endogenous CHS gene in the genetically modified flax

**Figure 2:**
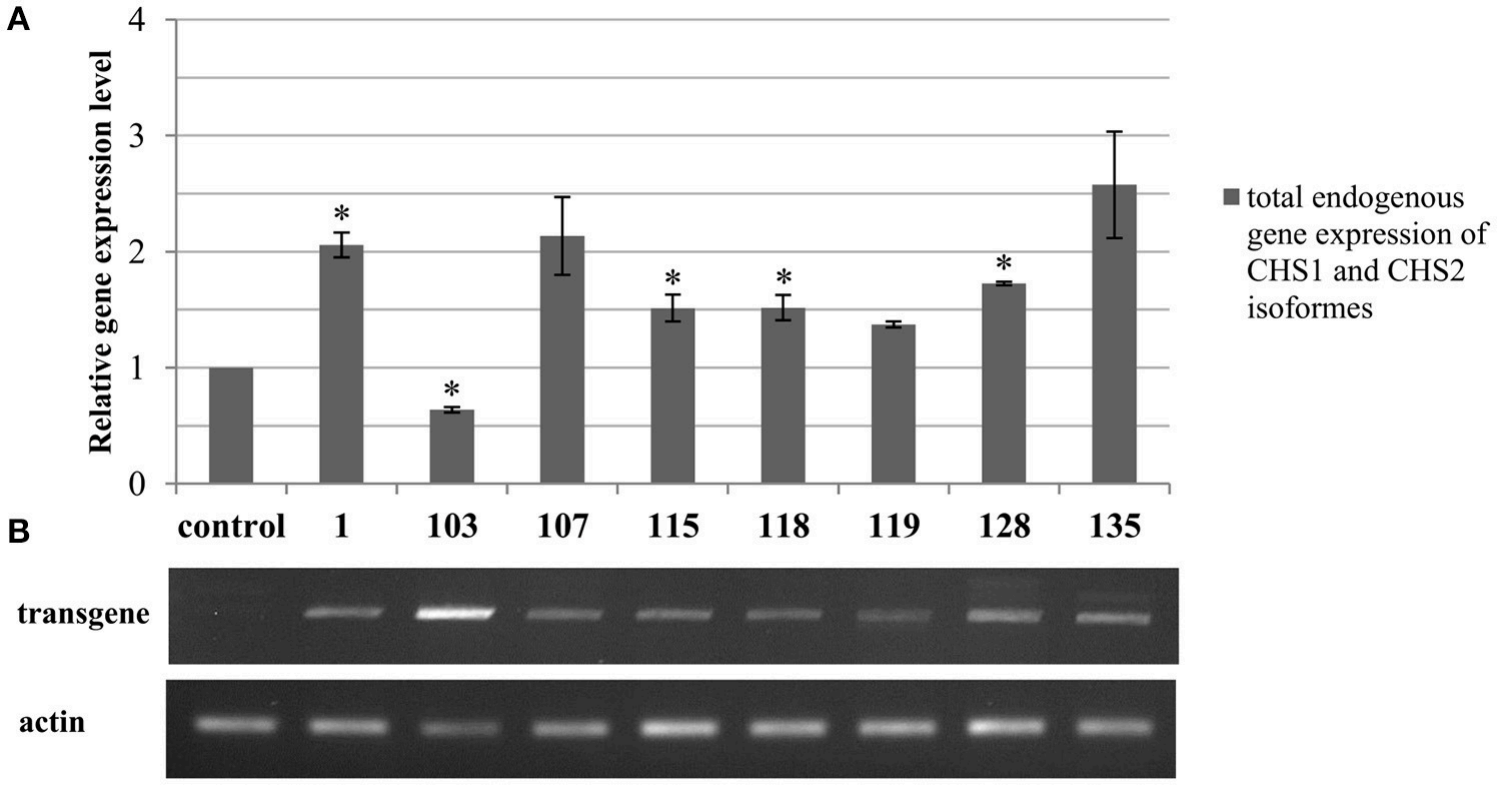
**Analysis of the total endogenous CHS isoforms (***CHS1*** and ***CHS2***) gene expression (A)** and transgene expression in transgenic flax **(B). (A)** In the chart transcript levels are normalized to the levels observed in the control plants (set as 1). Each value is presented as the relative gene expression level. Data constitute the mean value ± SD from at least three technical repeats of three independent biological samples. The significance of the differences between each mean and control was determined by Student's *t*-test. Asterisk indicates *p* < 0.05. **(B)** The expression level of the synthase chalcone transgene in the genetically modified flax with *CHS* cDNA from *Petunia* x *hybrid* is presented as the products of semi-quantitative PCR separated in the gel electrophoresis. The original picture of the gel electrophoresis along with the molecular weight marker is presented in the Supplementary Figure 3.

The level of mRNA derived from the endogenous CHS gene was determined in the transgenic plants. The analysis showed highly variable expression, either overexpression or repression in comparison to the control plants (Figure 2A). Mostly, the transgenic lines showed overexpression of the endogenous gene. In lines 107, 128, and 135 it was approximately 2-fold higher in comparison to the wild type Linola flax. Also transgenic lines 115, 118, 119, and 128 possessed an elevated transcript level of the endogenous CHS gene, but the relative gene expression levels values varied between 1.37 and 1.73. Only transgenic line 103 presented decreased expression of the endogenous gene (almost by half) in comparison to the control plants.

For the further analysis there were selected three lines: 115, 118, and 128. Although the other plants presented significant differences in comparison to the control plants, the selected lines presented the most stable changes in time (at least two generations).

#### Analysis of transgene expression

Expression of the transgene was identified by using a primer specific only to the exogenous gene. The expression levels differed between different lines. The major expression level was observed in line 103, which was increased the most among obtained plants. The highest values were also observed in lines 107, 128, and 135. In comparison to other transgenic plants the lowest expression level was noted in 119, which was significantly lower in comparison to line 103 (Figure 2B).

### ODN application

#### Design of ODN sequences homologous to the endogenous CHS gene

In order to design the short oligonucleotides homologous to the chalcone synthase gene sequence, the Mfold software was used; it predict the most suitable sites in RNA for ODNs to bind, primarily on the basis of the nucleotide composition (%GC) and the potential secondary structure of the short oligonucleotide. For the endogenous flax CHS gene 11 ODNs were designed and synthesized. The sequences were homologous to the following regions: 5′-UTR (ODNs 1 and 2), intron (ODNs 5 and 6), coding sequence (exon 1: ODNs 3–4, exon 2: ODNs 7–10) and the 3′-UTR region (ODN 11). The sequences were mostly homologous to both isoforms. ODNs 5 and 6 were homologous only to *CHS2* (Table 1).

**Table 1:** **ODNs in the antisense orientation designed for CHS gene sequences**.

**Region**	**ODN**	**Locus**	
		***CHS1***	***CHS2***
5′UTR	1	−133	−136
	2	−96	−99
Exon1	3	+1	+1
	4	+57	+57
Intron	5	–	+266
	6	–	+665
Exon2	7	+471	+915
	8	+606	+1,050
	9	+667	+1,110
	10	+1,003	+1,450
3′UTR	11	+1,371	+1,824

#### Total expression of the endogenous CHS gene in ODN-treated flax

**Figure 3:**
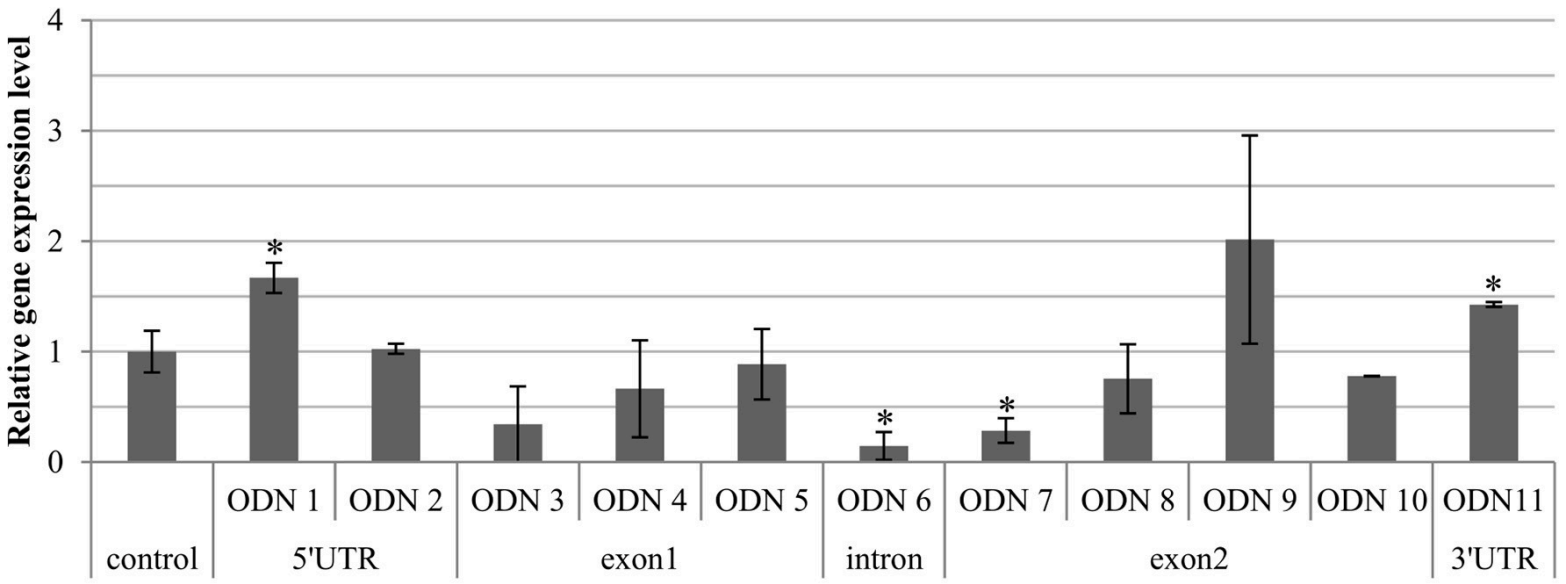
**Expression level of total endogenous CHS gene isoforms (***CHS1*** and ***CHS2***) in the flax treated with ODNs**. The level of the transcript was determined in the material from plants at 24 h after exposure to ODNs, in comparison to the control. The data are the result of the real-time PCR analysis. The reference gene was actin. mRNA levels were normalized to the levels observed in the control plants (set as 1). Each value is presented as relative gene expression level. Data constitute the mean value ± standard deviation (SD) from at least three technical repeats of three independent biological samples. The significance of the differences between each mean and control was determined by Student's *t*-test. Asterisk indicates *p* < 0.05.

The expression level of the endogenous CHS gene 24 h after treatment with ODN varied in the plants and exhibited both overexpression and repression, in comparison to the control plants (Figure 3). This event was also primarily observed in the GM-CHS flax. The flax treated with ODNs 1, 9, and 11 showed at least a 1.5-fold increase in the transcript level, whereas the majority of the remaining sequences led to repression of the endogenous CHS gene. The most decreased values were observed in ODNs 6 and 7 (0.15 and 0.28, respectively). From the range of the ODN-treated plants, there were selected three that were characterized by the most visible and significant modulation of the CHS gene: ODNs 1, 6, and 11, localized in the regulatory regions.

#### Stability of total CHS expression in time in ODN-treated plants

**Figure 4:**
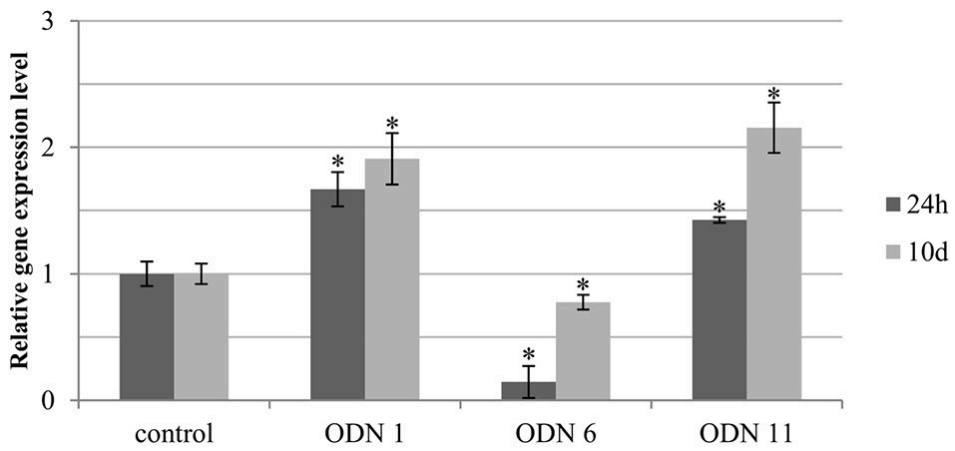
**Expression level of total endogenous CHS gene isoforms (***CHS1*** and ***CHS2***) at various time points in selected plants treated with ODNs**. The level of the transcript was determined in the material from plants at 24 h (dark gray) and 10 days (light gray) after exposure to ODNs, in comparison to the control. Transcript levels were normalized to the levels observed in the control plants (line in bold, set as 1). Each value is presented as relative gene expression level. Data constitute the mean value ± SD from at least three independent experiments. The significance of the differences between each mean and control was determined by Student's *t*-test. Asterisk indicates *p* < 0.05.

In plants treated with the selected ODNs (ODNs 1, 6, and 11) the expression of the endogenous chalcone synthase gene at various time points was determined. We observed that after the time of 10 days, in all three ODNs the CHS gene expression was elevated, compared to the previous time points. However, the general tendency of the changes was maintained (Figure 4).

The introduced transgene (*Petunia hybrida* chalcone synthase gene) indicated similar homology to both analyzed CHS isoforms, but some ODNs used for epigenetic modification—such as ODN 6—show specificity to one isoform (CHS2), so it makes sense to measure expression of the two isoforms separately.

### Expression of *CHS* isoforms

**Figure 5:**
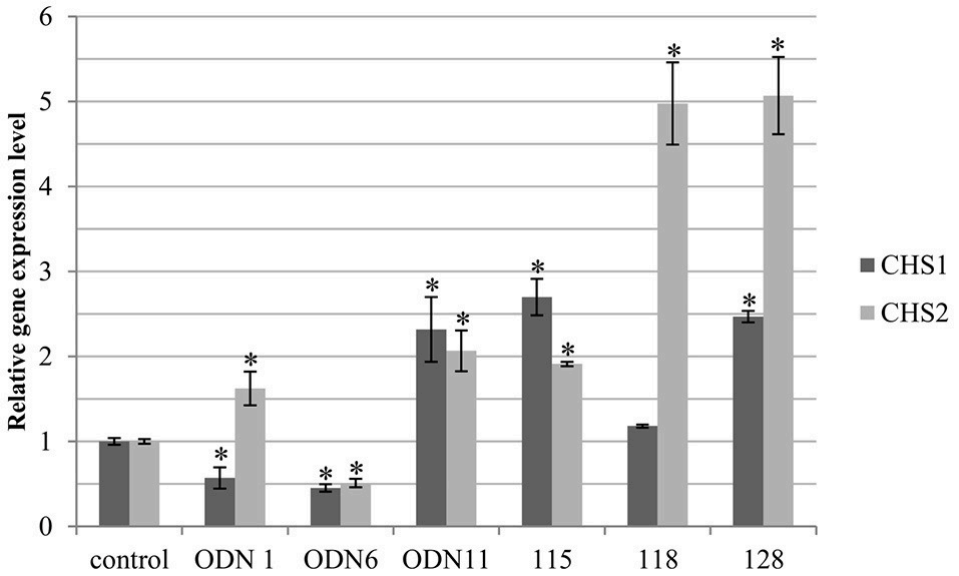
**Expression levels of CHS gene isoforms (***CHS1*** and ***CHS2***)**. The level of the isoforms transcript was determined in the material from plants at 24 h after exposure to ODNs and in *in vitro* cultured transgenic lines. The figure presents expression levels of *CHS1* (dark gray) and *CHS2* (light gray). Transcript levels were normalized to the levels observed in the control plants (set as 1). Each value is presented as relative gene expression level. Data constitute the mean value ± SD from at least three independent experiments. The significance of the differences between each mean and control was determined by Student's *t*-test. Asterisk indicates *p* < 0.05.

In the selected flax treated with ODNs and genetically modified, the expression of two *CHS* isoforms, *CHS1* and *CHS2*, was analyzed. In the genetically modified flax the transcript level of both isoforms was mostly elevated. The CHS1 gene was significantly increased in line 115 and 128 (by 170 and 146%, respectively). All three analyzed transgenic lines showed intense elevation of the *CHS2* isoform: 2-fold in line 115 and 5-fold in lines 118 and 128. According to the flax treated with ODNs the transcript level of *CHS1* was significantly increased (by 132%) in ODN 11 and reduced in ODN 1 and ODN 6 (by 43% and 55%, respectively). The expression of the *CHS2* isoform was changed proportionally to the total *CHS* expression in all three plants. In comparison to the control plants, in ODNs 1 and 11 the expression was higher (by 62 and 106%, respectively), whereas in the flax treated with ODN 6 the expression was decreased by almost 50% (similarly to the *CHS1* gene expression) (Figure 5).

### Total methylation of genomic DNA

Methylation is one of the most common mechanisms of epigenetic modification in plants. Since genetic modification may also indicate changes in general methylation, or methylation of the target gene, the level of methylation was measured in transgenic and ODN-treated plants. Such comparison of transgenic plants and plants treated with ODN is a good tool to determine whether the transitional presence of ODN sequences homological to the endogenous gene may have a similar effect (in indication of methylation changes) as stable genetic transformation.

**Figure 6:**
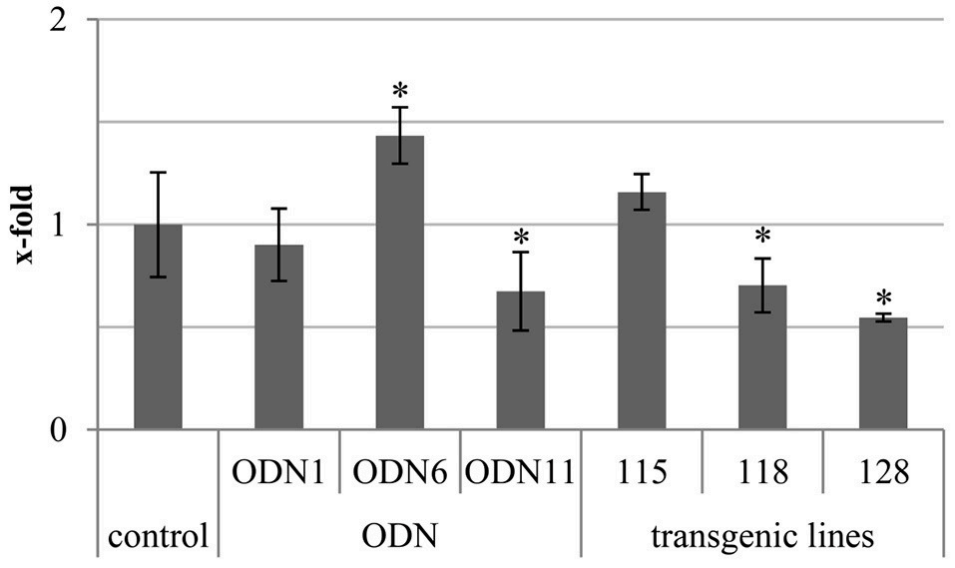
**Total genomic methylation in ODN-treated and GM flax**. The total genomic methylation was determined in the genomic DNA isolated from material collected at 48 h after exposure to ODNs and from *in vitro* cultured transgenic lines. The experiment was performed using a technical kit dedicated for total methylation assay (detailed description in Materials and Methods section). Data constitute the mean value ± SD from at least three independent experiments. The significance of the differences between each mean and control was determined by Student's *t*-test. Asterisk indicates *p* < 0.05.

The total genomic methylation was monitored by the level of 5-methylated cytosines in the genomic DNA. The measurements were performed on the plant material collected 48 hrs after treatment. The total methylation in the genomic DNA was greatly different in comparison to the control plants in both genetically modified and ODN-treated flax. The greatest increase was observed for ODN 6 and line 115. However, the observed increase for line 115 was not statistically significant. A reduction was observed in ODN 11 (0.67) and line 128 (0.55). In many cases, especially in ODN-treated plants, there was a negative correlation between CHS gene expression and total methylation of the genome (Figure 6).

### Pattern of DNA methylation in specific regions of the CHS gene

**Figure 7:**
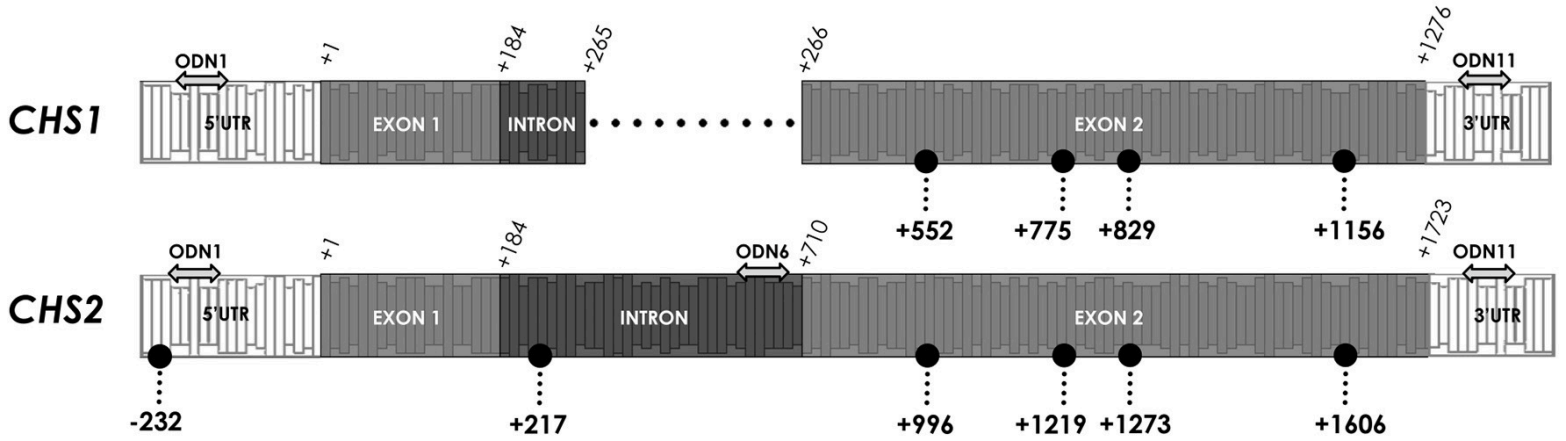
**Crucial CCGG sites in CHS gene isoforms**. The color of dots indicates different regions and changes in character of the CCGG site. The location of the CCGG in sequences of *CHS1* and *CHS2* are in brackets (*CHS1*/*CHS2*). Stably demethylated sites: 5′-UTR (—/−232), non-coding (—/+217) and coding (+1,156/+1,606). Variable sites: coding region (+552/+996, +775/+1,219, +829/+1,273). The double-headed arrows indicate the particular ODNs used in the experiment: ODN 1 (−133/−136), ODN 6 (—/+665) and ODN 11 (+1,371/+1,824).

In order to determine the characteristic pattern of CHS gene methylation, crucial -CCGG- motifs in the CHS gene sequence were analyzed. Among studied sites, stable demethylated and highly variable in methylation -CCGG- sites were observed. Stable lack of cytosine methylation was observed in the following regions: 5′-UTR (−232), non-coding (+217) and exon 2 (+1,606), which were basically unchanged in comparison to the control plants. Variable sites were noted only in the coding region (+996, +1,219, +1,273). The variability of sites indicates the differences in the methylation of single inner cytosine and both cytosines (Figure 7).

**Figure 8:**
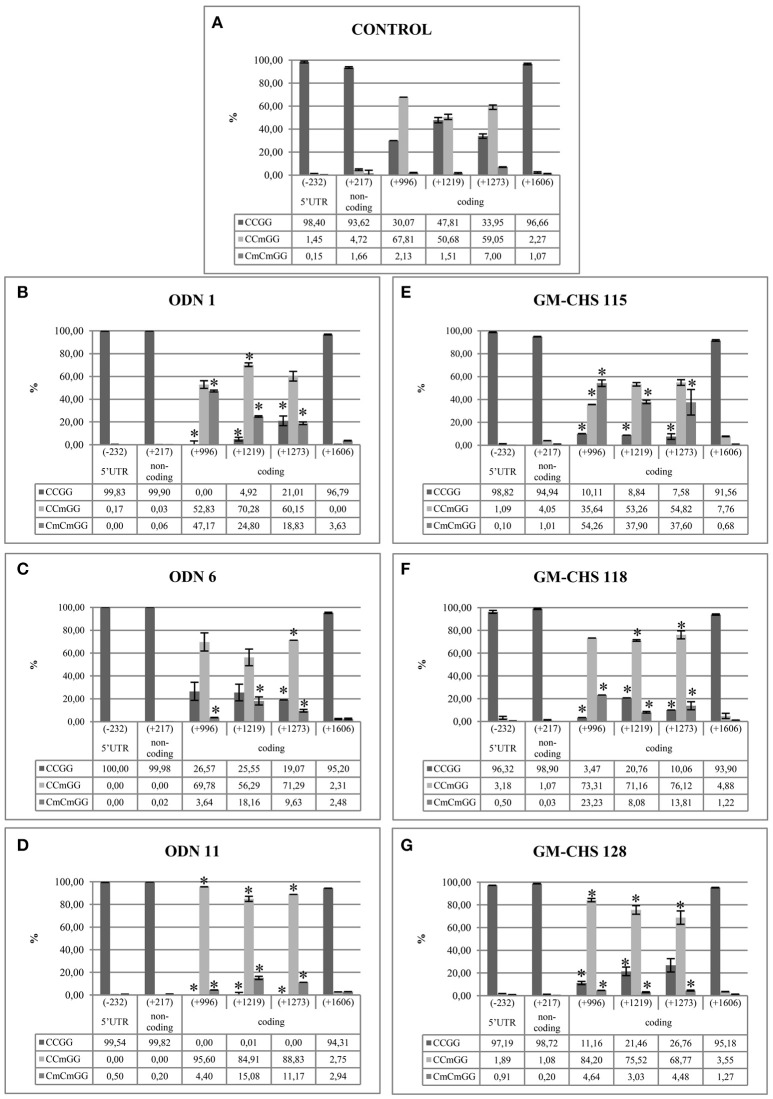
**Stable demethylated and variable methylation profiles for the control (A)**, ODN-treated **(B–D)** and GM-CHS **(E–G)** plants. The figure presents the percentage of the cytosine methylation in the CCGG sites in the flax genome. The genomic DNA was digested by restriction enzymes HpaII and MspI. The amount of non-digested DNA was determined by real-time PCR. The percentage of particular modification was presented for studied plants and control: CCGG, lack of methylation (dark gray); CCmGG, methylation of internal cytosine (light gray); CmCmGG, methylation of both cytosines (middle gray). The site positions were presented according to the *CHS2* sequence (the presence of all CCGG sites). Stably demethylated sites: 5′-UTR (−232), non-coding (+217) and coding (+1,606). Variable sites: coding region (+996, +1,219, +1,273). Data constitute the mean value ± SD from at least three independent experiments. The significance of the differences between each mean and control was determined by Student's *t*-test. Asterisk indicates *p* < 0.05.

In the ODN-treated flax in all variable sites the total level of methylation was elevated in comparison to the control plants. The methylation level of two cytosines was the highest in ODN 1, especially at site +996 (increase by 45%). However, the most elevated level of single cytosine methylation was noted in ODN 11, by ~30% in all three sites. The methylation profiles of flax treated with ODN 6 were mostly similar to the control plants (Figure 8).

The -CCGG- motifs (−232, +217, and +1,606), which presented stable demethylation in the ODN-treated plants, were also demethylated in GM flax. The total methylation was increased in the genetically modified plants in comparison to the control plants. At all three sites the major changes were noted for line 115, where the methylation level of both cytosines was increased by 52, 36, and 30%, respectively. In lines 118 and 128 the methylation of single cytosine was increased by ~20%, at sites +1,219 and +1,273 (Figure 8).

### Expression of EPI-genes (genes involved in epigenetic modifications)

**Figure 9:**
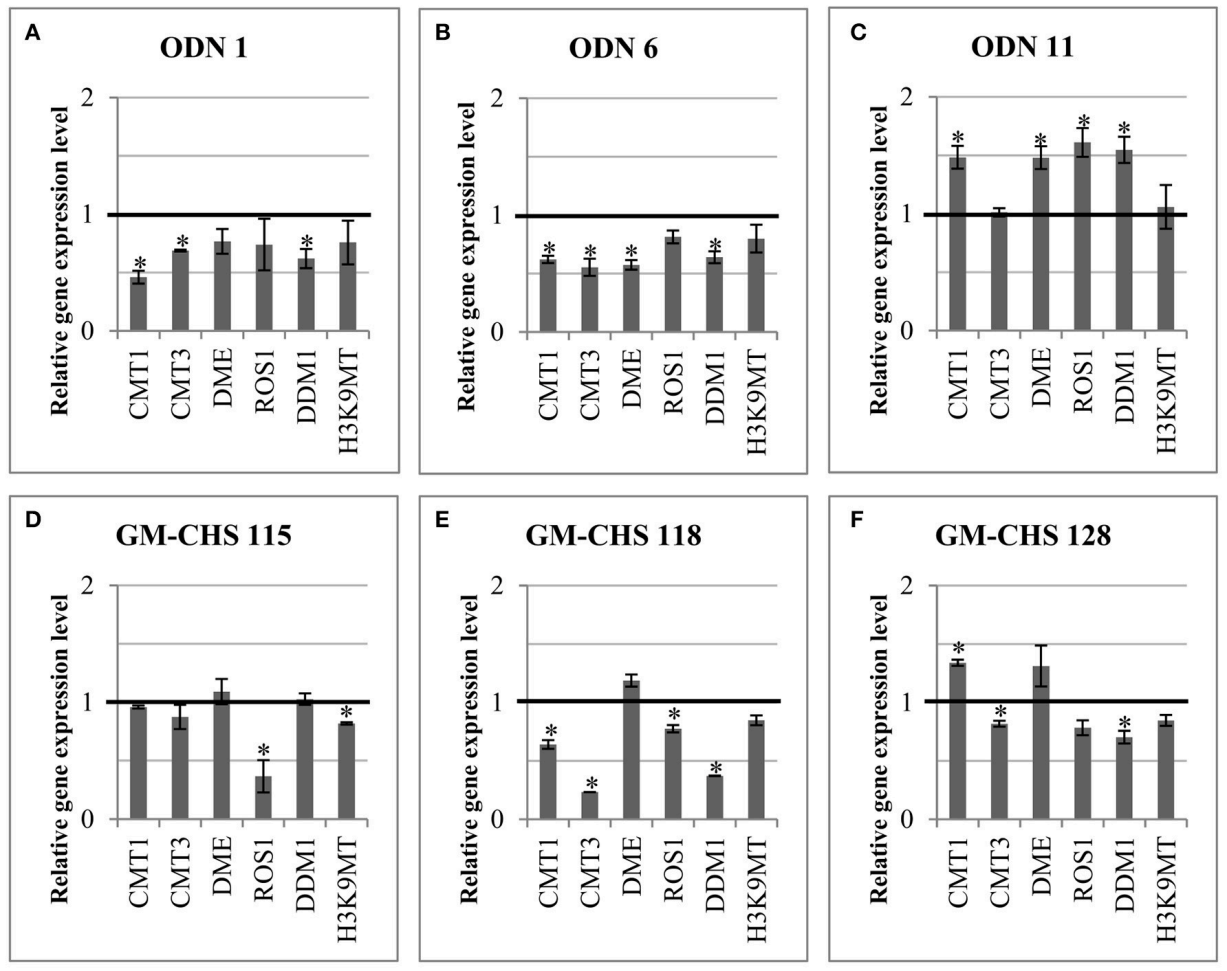
**Expression of genes involved in epigenetic modifications in ODN-treated (A–C)** and transgenic **(D–F)** plants. Expression of the methylases (*CMT1*, chromomethylase 1; *CMT3*, chromomethylase 3) and demethylases (*DME*, DEMETER; *ROS1*, repressor of silencing 1) and enzymes involved in chromatin methylation (*DDM1*, decrease in DNA methylation 1; *H3K9MT*, histone H3K9 methyltransferase) is presented for plants. The control is presented as a line in bold (set as 1). Data constitute the mean value ± SD from at least three independent experiments. The significance of the differences between each mean and control was determined by Student's *t*-test. Asterisk indicates *p* < 0.05.

In order to explain the mechanism of epigenetic modification, the expression of genes involved in the epigenetic modification (epi-genes) was investigated (Figure 9). Thus the gene expression of methylases (CMT1 – chromomethylase 1, CMT3 – chromomethylase 3), demethylases (DME – DEMETER, ROS1 – repressor of silencing 1) and enzymes involved in chromatin methylation (DDM1 – decrease in DNA methylation 1, H3K9MT – histone h3k9 methyltransferase) was analyzed.

In comparison to the control plants, minor changes were noted in flax plants at 24 hrs after exposure to ODNs (data not shown). However, after 48 h the transcript level of all epi-genes was altered in comparison to the control plants. ODN 1 and ODN 6 triggered the reduction of all epi-genes in flax; in particular, ODN1 decreased *CMT1* (to 46%) and *DDM1* (to 62%) and ODN 6 decreased *CMT1, CMT3* and *DME* (to 62, 55, 57%, respectively). In contrast, in ODN 11 overexpression of genes encoding methylases and demethylases was observed, except *CMT3* and *H3K9MT* (compared to the control). The other genes showed an increase in the transcript level by ~50%.

In the GM-CHS flax the expression of epi-genes was reduced less than in the plants exposed to ODNs 1 and 6. In 115 line the gene expression was similar to the control plants, except the significant reduction of *ROS1* (to 37%) and minor reduction of *H3K9* methyltransferase (to 81%). In all three transgenic plants the DME was non-significantly increased. In line 118 the transcript levels of both methylases and demethylases were reduced, especially *CMT3* (to 23%) and *DDM1* (to 37%). The 128 transgenic flax showed an increased level of *CMT1* (by 34%) and minor reduction by approximately 20% in the transcript level of other genes.

### *In silico* study

**Figure 10:**
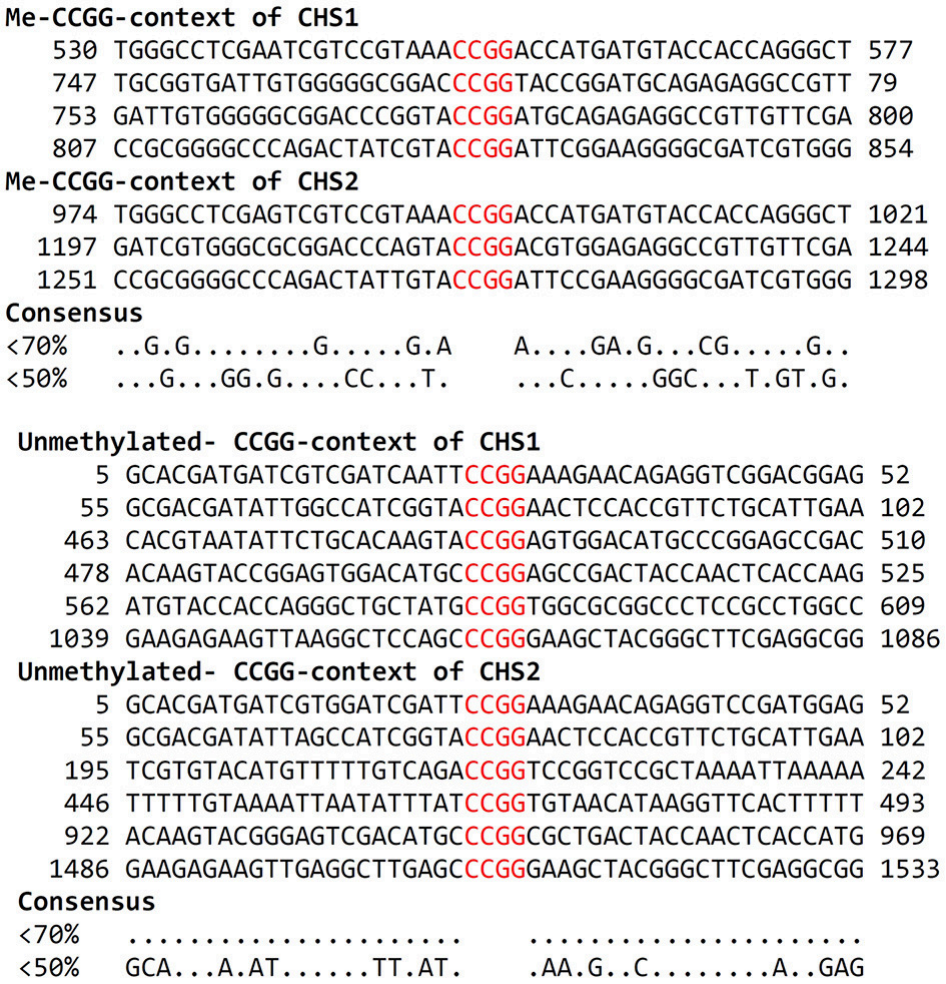
**Alignment of -CCGG- motifs context of CHS gene**. The nearest vicinity of methylated and unmethylated -CCGG- motifs in *CHS1* and *CHS2* isoforms in the flax genome was compared. According to each motif, 22 nucleotides upstream and downstream of the -CCGG- motifs were analyzed. For each -CCGG- motif, GC content (%) was calculated (presented on the left).

An important issue is the mechanism that discriminates -CCGG- motifs for methylation. One possible mechanism could be the nucleotide context. The comparative analysis of methylated and unmethylated -CCGG- motifs reveals that there is no strictly defined consensus sequence surrounding either of these motifs but the methylated ones are certainly enriched with GC pairs (Figure 10).

Another theory may involve changes to the methylated DNA conformation. To study this, the web server offers nucleosome stability model investigation. For example, the interactive chromatin modeling tool generates a nucleosome energy level diagram. Using this, the location of the nucleosome in DNA upon methylation was predicted, and the respective diagram is presented in Supplementary Figure 1.

It can be clearly seen that C to T changes in -CCGG- motifs result in an energy increase and the nucleosome sliding toward the DNA 3′-end. Fortunately this DNA region contains sites recognized by AatI and PvuI restriction enzymes, thus making it possible to probe this sequence in the direction of structural changes. For that reason DNA isolated from control plants and another transgenic plant with stable epimutation (5th generation) was probed with restriction enzymes followed by PCR (Supplementary Figure 2).

It can be clearly seen that DNA from control and epimutated plants significantly differs in accessibility to restriction enzymes. Thus, it is suggested that methylation provokes elastic DNA deformations that match the nucleosome' geometry. The suggestion requires, however, further investigation.

## Discussion

The role of epigenetics in plant genome diversification and thus evolution is quite obvious. Indeed, the recent results on the comparable analysis of ancient and modern pea DNAs reveal several nucleotide substitutions, and in the majority two types of transitions, C to T and G to A, have been detected (Smýkal et al., 2014). Such substitutions might result from deamination of 5-methyl cytosine to thymine, which is typical for epigenetic changes.

In determining how epigenetics acts, we concentrate on the integration of gene expression and gene-body methylation, the latter induced by two signals, ODNs and transgenesis. It was recently highlighted that the ODN technique can be a rapid and time-serving antecedent in quick analysis of gene function as vector-mediated transformation because of the fact that both silence the gene with the same efficiency and produce a similar phenotype effect (Liao et al., 2013). It is hypothesized that ODNs can down-regulate the gene through the mechanism collectively known as RNA interference (RNAi) (Baulcombe, 2004). Recently it has also been shown that small exogenous DNA fragments might activate homologous genes, if they are targeted to the regulatory sequences. This new mechanism, termed RNA activation (RNAa), ascribed previously only to mammalian cells, was very recently detected also in plants (Hollister and Gaut, 2009).

In our study, in most transgenic lines, stable overexpression of the endogenous CHS gene was observed.

The treatment of flax with a series of short oligonucleotides homologous to a different part of CHS gene isoforms revealed that those directed to regulatory gene regions (5′- and 3′-UTR) activate gene expression, directed to non-coding region (introns) caused gen activity reduction, while those homologous to a coding region have a variable effect but mostly suppressed its activity. The possible explanation of gene silencing may be that sequences complementary to mRNA of the target gene would cause RNase H cleavage and inhibition of mRNA transcription (Giles and Tidd, 1992), or formation of a complex (a triplex with one DNA strand that affects RNA polymerase walking as described by Joseph et al. (1997). More interesting is how ODNs homologous to 5′- and 3′-UTR activate the gene. According to our results, a possible explanation is that gene expression changes were accompanied by changes in its methylation status. But only certain -CCGG- motifs along the gene sequence were affected. It is noted that the changes in methylation concern the same motifs no matter what ODN is analyzed. This might suggest that the nucleotide sequence around -CCGG- motifs and thus its secondary structure “filters” methylation accessibility. The comparative analysis of the nucleotide of methylated and unmethylated motifs showed that there are only a few positions that are unique for methylated and unmethylated motifs. However, the sequence surrounding (22 bp in both 5′- and 3′- directions) methylated motifs is enriched with GC content. This might suggest that the sequence context affect motifs' accessibility for the methylation machinery.

Further analysis reveals that methylated motifs are located within CpG islands. The islands have been identified in many eukaryotic organisms, and in vertebrates they remain unmethylated. It is believed that prevention of methylation is an intrinsic property of these. For example, the human CpG introduced into *A. thaliana* retained all features of human motifs (Meza et al., 2002). It is evidenced that CpG islands also exist in plants (Ashikawa, 2001). However, plants exhibit a mosaically methylated genome comprising interspersed methylated and unmethylated regions covering whole gene regions (Feng et al., 2010).

It is interesting that CpG in the CHS gene reveals discrete heterogeneity. The same ODN induces a different methylation profile in three analyzed -CCGG- motifs of the CpG cluster, which suggests their differential accessibility. Indeed, the gene expression profile responds to the methylation level. ODNs directed to 5′- and 3′-UTR (1 and 11) produce a significant increase in mRNA synthesis concomitant with the high level of methylated C in the three analyzed -CCGG-motifs, whereas that directed to the intron (ODN 6) shows a rather slight methylation increase which is however reasonable for a decrease of gene expression. In vertebrate CpG islands there appears to be a relatively homogeneous fraction of the genome, although heterogeneity of individual nucleotide sequences was detected (Deaton and Bird, 2011).

It was expected that methylation of DNA would affect its ability to form nucleosomes and eventually would lead to distinctive nucleosome positioning patterns in a gene. For that reason, an interactive chromatin modeling web server tool has been used for generating a nucleosome energy level diagram and nucleosome placing in the energy landscape (Stolz and Bishop, 2010). The calculation predicts that high-energy, less stable nucleosomes may be formed when the DNA sequence exhibits C to T changes at the sites determined experimentally (HpaII-MspI digestion). The DNA region with predicted sequence elastic deformation upon methylation was probed with two restriction enzymes. Type II restriction endonuclease cleavage occurs at a precise DNA sequence, but the level of enzyme activity is also regulated by the sequence context of the enzyme binding site (Wei et al., 2008). It was thus assumed that probing the DNA with restriction enzymes would provide the information on its deformation upon methylation. AatII and PvuI digestion followed by PCR reveals significant differences between the control and methylated DNA. Thus, the default energy model exhibits a correlation with experimentally determined DNA deformation upon methylation.

In order to investigate the stability of *CHS* epimodulation, we measured time-dependent gene expression in plant treated with ODNs and the methylation profile in plants transformed with *Petunia hybrida CHS* homolog in sense orientation. The results obtained suggest that methylation and thus gene expression is a dynamic process. Initial changes in gene activity could revert to the control level, and thus long-term investigation is suggested. Of interest was the analysis of the methylation profile in transgenic plants. All three selected transgenic lines showed changes in the methylation profile of -CCGG- motifs located within a CpG island. The profile was very similar to those induced by ODNs directed to 3′- and 5′-UTR. Thus, genetic signals such as stable transgenes and epi-signals such as ODN produce the same and, more important, heritable effect, with respect to methylation of their homologous gene.

An important aspect of epi-mutation is expression analysis of genes participating in metabolism of the DNA cytosine methyl residue. Three categories of methylated cytosine sequence context can be recognized in plants: CG, CHG, and CHH (H = A, C, or T). Their methylation is maintained by the methyltransferase enzymes MET1, CMT1, and CMT3, respectively. The glycosylase enzymes such as ROS1 and DME are involved in cytosine demethylation.

The level of these epi-genes' expression was determined using RNA derived from the control plants as well as from plants treated with ODN and GM flax. By employing this analysis it was found that both methyltransferase and demethylase mRNA levels significantly decrease, but in most plants the ratio between them was close to the control. It thus suggests that -CCGG- motifs methylation induced by ODNs derives from greater accessibility of the methylated region rather than activation of respective enzymes. In the case of transgenic plants, both methyltransferases and *ROS1* glycosylase gene expression varied dependently on the line. The results showed that there is no simple correspondence between DNA methylation and the expression of the genes involved in the methylation mechanism. However, we observed in the majority of analyzed plants that in the relation to the total methylation level, DME and ROS1 are presumably the most prominent epi-genes, which reflect the changes in methylation. Interesting to note is the lowered expression of the histone H3K9 methyltransferase gene, while histone acetyltransferase gene expression was at the control level. It thus suggests that ODNs also affect the methylation status of histone via a general mechanism involving respective gene expression. The reason for this is as yet unknown.

Inducible response genes fall into two classes: those that require a chromatin remodeling complex (e.g., SWI/SNF) and those that do not. It was evidenced that genes with a CpG island in the regulatory region showed intrinsic accessibility and do not need to be remodeled (Deaton and Bird, 2011). The analysis of the SWI/SNF homolog, DDM1, showed expression reduction in both ODN-treated and transgenic plants. This might suggest that CHS gene expression changes upon ODN treatment are due to methylation profile changes and thus nucleosome structure rearrangement within the CpG gene region.

In summary, the acquired data reveal that the methylation status of plants upon ODN treatment as well as transgenesis is changed. The changes concern the same -CCGG- gene motifs and resulted in gene activity modification. It thus suggests the epigenetic-genetic interaction in gene expression regulation mediated by DNA methylation. The analyzed DNA motifs (corresponding to three categories of cytosine methylation in plants) of the flax CHS gene are more accessible for methylation when located within a CpG island. The motifs methylation resulted in rearrangement of nucleosome location and thus gene expression changes. However, another mechanism such as competition for transcription factors between ODN and its gene homolog cannot be excluded. It is also important to note that ODN treatment induces heritable genome diversification and might be a valuable alternative for improvement of crops.

## Author contributions

MD and MZ conceived and designed the experiments, analyzed the data and wrote the manuscript, MD performed the experiments, JS and MZ supervised the work and revised the manuscript, TC performed the bioinformatic *in situ* analysis. All authors reviewed and approved the manuscript.

### Conflict of interest statement

The authors declare that the research was conducted in the absence of any commercial or financial relationships that could be construed as a potential conflict of interest.

## Supplementary material

Supplementary Figure 1 **Energy landscape and nucleosome footprint**. Energy level (kcal/mol nucleotide) diagram of CHS2 gene and predicted nucleosome (NS) location upon methylation. Blue line and bars, control DNA and NS; red line and bars, methylated DNA and NS. Green bars indicate exons and yellow CpG island. Also the positions of -CCGG- motifs affected by methylation (red) and not affected (blue) are included. The approximate locations of restriction sites of PvuI and AatII enzymes for DNA accessibility for restriction analysis are marked as purple lines.

Supplementary Figure 2 **DNA accessibility for restriction**. DNA from nontransgenic (Linola) and GM flax (W.92) lines with stable modulation of CHS gene expression) treated with restriction enzymes was followed by real-time PCR. The level of products defines DNA accessibility for restriction. The Linola flax was set as 1 (control) for each restriction enzyme.

Supplementary Figure 3 **Original image of PCR products separated in gel electrophoresis presented in Figure 2**. Upper row, actin; lower row, transgene. Order of samples in each row: marker, control, GM-CHS: 1, 103, 107, 115, 118, 119, 128, 135. The legend on the left presents the size of the bands from the molecular weight marker. The size of PCR products is presented on the right.

Table S1 **Sequences of designed ODNs and their parameters (length, %GC, location)**.

Table S2 **Sequences of primers used for real-time PCR analyses**.

## Abbreviations

A: adenine
BAP: 6-benzylaminopurine
bp: base pair
C: cytosine
cDNA: complementary DNA
CHS: chalcone synthase
CIM: callus inducing medium
Cm: methylated cytosine
CMT1: chromomethylase 1
CMT3: chromomethylase 3
CpG: cytosine poly-guanine site
DDM1: decrease in DNA methylation 1
DME: demeter
dsRNA: double stranded RNA
G: guanine
GM: genetically modified
GMO: genetically modified organism
H, adenine: cytosine or thymine
H3K9MT: histone H3K9 methyltransferase
mRNA: messenger RNA
MS: Murashige-Skoog
MET1: DNA (cytosine-5)-methyltransferase 1
NAA: 1-naphthaleneacetic acid
NS: nucleosome
nt: nucleotides
OCS: octopine synthase
ODNs: oligodeoxynuleotides
PCR: polymerase chain reaction
PPM™: plant preservative mixture
RdRP: RNA-dependent RNA polymerase
RNAa: RNA activation
RNAi: RNA interference
ROS1: repressor of silencing 1
RQ: relative quantity
SBE: starch branching enzyme
shRNA: short hairpin RNA
SIM: shoot inducing medium
siRNA: small interference RNA
SWI/SNF: chromatin-remodeling complex SWItch/Sucrose Non-Fermentable
T: thymine
TE: transposable element
Tm: melting temperature
UTR: untranslated region
UV: ultraviolet radiation.
