# Supplementary figures and images for: Oligodeoxynucleotides Can Transiently Up- and Downregulate CHS Gene Expression in Flax by Changing DNA Methylation in a Sequence-Specific Manner
Source: Front Plant Sci. 2017 May 15;8:755. doi: 10.3389/fpls.2017.00755 (PMC5430052; doi:10.3389/fpls.2017.00755)

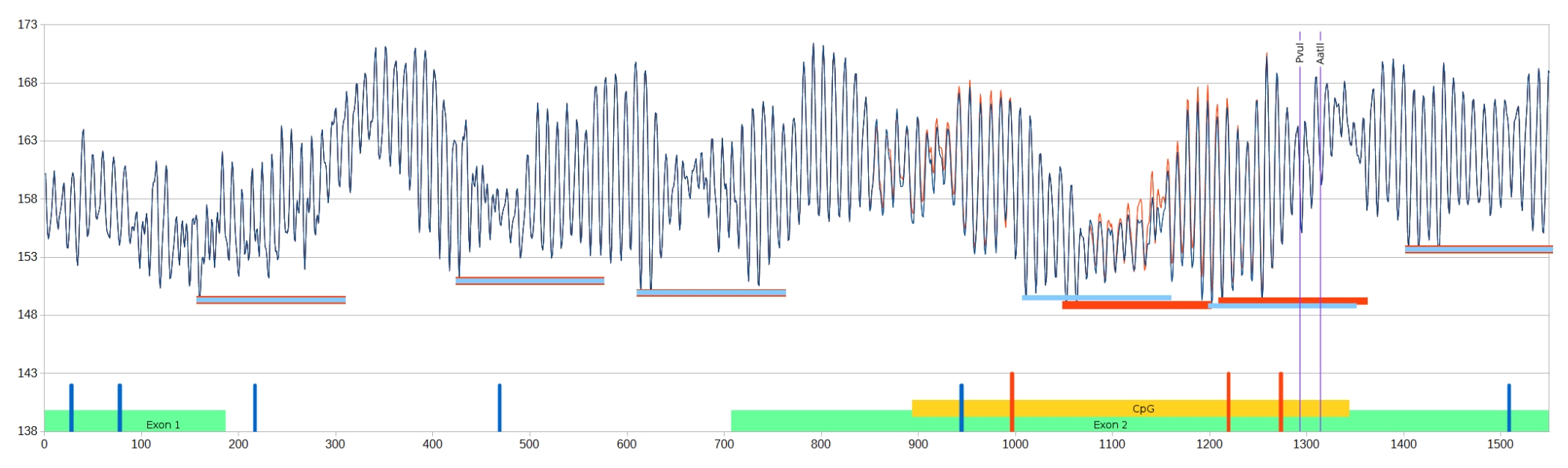

Supplement: Supplementary Figure 1 — Energy landscape and nucleosome footprint. Energy level (kcal/mol nucleotide) diagram of CHS2 gene and predicted nucleosome (NS) location upon methylation. Blue line and bars, control DNA and NS; red line and bars, methylated DNA and NS. Green bars indicate exons and yellow CpG island. Also the positions of -CCGG- motifs affected by methylation (red) and not affected (blue) are included. The approximate locations of restriction sites of PvuI and AatII enzymes for DNA accessibility for restriction analysis are marked as purple lines. [file Image1.JPEG]

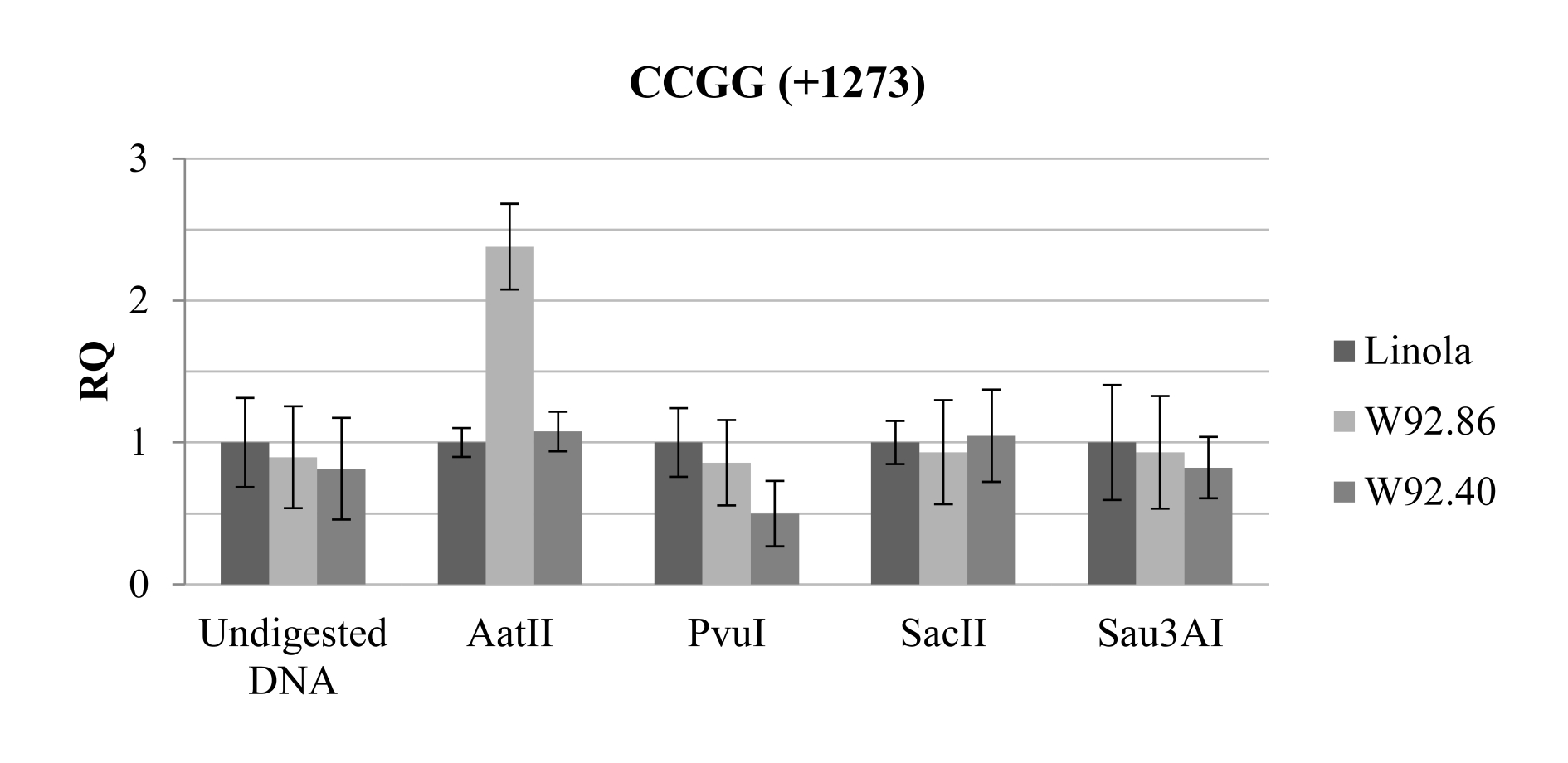

Supplement: Supplementary Figure 2 — DNA accessibility for restriction. DNA from nontransgenic (Linola) and GM flax (W.92) lines with stable modulation of CHS gene expression) treated with restriction enzymes was followed by real-time PCR. The level of products defines DNA accessibility for restriction. The Linola flax was set as 1 (control) for each restriction enzyme. [file Image2.JPEG]

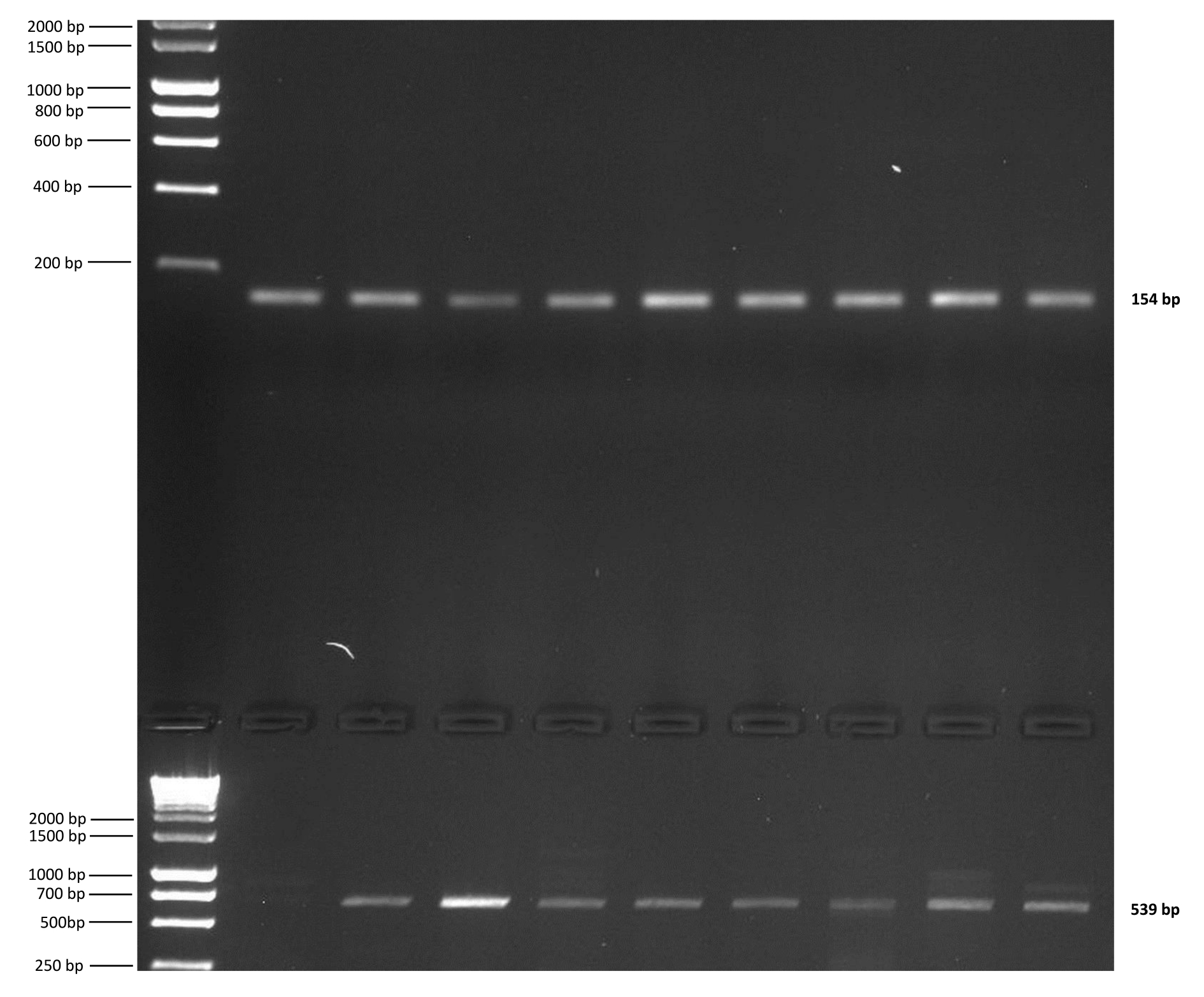

Supplement: Supplementary Figure 3 — Original image of PCR products separated in gel electrophoresis presented in Figure 2. Upper row, actin; lower row, transgene. Order of samples in each row: marker, control, GM-CHS: 1, 103, 107, 115, 118, 119, 128, 135. The legend on the left presents the size of the bands from the molecular weight marker. The size of PCR products is presented on the right. [file Image3.JPEG]
