# Supplementary material for: Oligodeoxynucleotides Can Transiently Up- and Downregulate CHS Gene Expression in Flax by Changing DNA Methylation in a Sequence-Specific Manner
Source: Front Plant Sci. 2017 May 15;8:755. doi: 10.3389/fpls.2017.00755 (PMC5430052; doi:10.3389/fpls.2017.00755)
Supplement: Table S1 — Sequences of designed ODNs and their parameters (length, %GC, location). [file Table1.DOCX]

**TABLE S1 Sequences of designed ODNs and their parameters (length, %GC, location).**

| **ODN (No.)** | **ODN sequence** | **Length [nucleotides]** | **%GC** | **Locus** | |
| --- | --- | --- | --- | --- | --- |
|  |  |  |  | ***CHS1*** | ***CHS2*** |
| 1 | 5' TACTGTCGGCTGACAGCT 3' | 18nt | 55,6% | -133 | -136 |
| 2 | 5' AATGGTGCATATATACCA 3' | 18nt | 40,0% | -96 | -99 |
| 3 | 5' ATGGGCACGATGATCGTC 3' | 18nt | 61,1% | +1 | +1 |
| 4 | 5' GCGACGATATTGGCCA 3' | 16nt | 38,9% | +57 | +57 |
| 5 | 5' TTGAACCATTAAAACC 3' | 16nt | 50,0% | - | +266 |
| 6 | 5' TCCGCTATTATCCATCGA 3' | 18nt | 44,5% | - | +665 |
| 7 | 5' ATTCTGCACAAGTACGGGAGT 3' | 21nt | 50,0% | +471 | +915 |
| 8 | 5' GGCCAAGGATCTGGCCGA 3' | 18nt | 50,0% | +606 | +1050 |
| 9 | 5' TACCACAATCGCATTTC 3' | 17nt | 72,2% | +667 | +1110 |
| 10 | 5' GCCCACCCTGGTGGGCCT 3' | 18nt | 77,8% | +1003 | +1450 |
| 11 | 5' ATGAAGATTAAATGTCCC 3' | 18nt | 40,0% | +1371 | +1824 |
