# Supplementary material for: Oligodeoxynucleotides Can Transiently Up- and Downregulate CHS Gene Expression in Flax by Changing DNA Methylation in a Sequence-Specific Manner
Source: Front Plant Sci. 2017 May 15;8:755. doi: 10.3389/fpls.2017.00755 (PMC5430052; doi:10.3389/fpls.2017.00755)
Supplement: Table S2 — Sequences of primers used for real-time PCR analyses. [file Table2.DOCX]

**TABLE S2 Sequences of primers used for Real Time PCR analyses.**

| **Target gene or sequence** | | **Primer forward** | **Primer reverse** |
| --- | --- | --- | --- |
| actin | | 5' CCGGTGTTATGGTTGGAAT 3' | 5' TGTAGAAAGTGTGATGCCAAA 3' |
| **CHS gene expression** | | | |
| *CHS* (total) | | 5' CCCACGTAATATTCTGCACAAGTA 3' | 5' GCGCCTCGATTGTTCTC 3' |
| *CHS1* | | 5' GCCATTGGGGGATATTGTTGG 3' | 5' CTTCCCGGGCTGGAGCCTT 3' |
| *CHS2* | | 5' ACCATTGGGGGATAATATTGTTGG 3' | 5' CTTCCCGGGCTCAAGCCTC 3' |
| **-CCGG- motif (*CHS1*/*CHS2*)** | | | |
| 5’UTR | (---/-232) | 5' CGAATCTCCCAACCTATCCA 3' | 5' GCTGGCCGTAAATAGTGCAT 3' |
| non-coding | (---/+217) | 5' AACATGGAACACAACACTCAGC 3' | 5' TTCAATGAACCCTGCACAAA 3' |
| coding | (+552/+996) | 5' ACCAACTCACCATGCTCTTG 3' | 5' GCGAAGTCGTCGTCACTAGG 3' |
|  | (+775/+1219) | 5' CGTAGTCTGGTGCCAGATCA 3' | 5' CTCGAACAACGGCCTCTCT 3' |
|  | (+829/+1273) | 5' GGGGCCCAGACTATTGTACC 3' | 5' CAATCACAATCGCCAACAAT 3' |
|  | (+1156/+1606) | 5' GGCGATTGTGATTGGAACTC 3' | 5' CCCATCTCCAGCAGACTTGT 3' |
| **The expression of genes encoding enzymes involved in the epigenetic modifications** | | | |
| *CMT1* | | 5’ CAGATTTCGCTCCACAGTA 3’ | 5’ AGAAATGTCCCATTGCTCTAT 3’ |
| *CMT3* | | 5’ AAAGGGTGCTAACTTCAGG 3’ | 5’ GACCAA ATGGTTTAGACGATGT 3’ |
| *DME* | | 5’ATGGCTACGGAGGCTACTTA 3’ | 5’ TGTTTCACCTGGTGTCCATA 3’ |
| *ROS1* | | 5’ GCACTGAGAAGAAGTGCC 3’ | 5’ CTTAATGCGTGCTGCAAG 3’ |
| *DDM1* | | 5’ GTGGTTAATATCTTTGTGGCAG 3’ | 5’ CATCCAATTTGCCAGAGTAG 3’ |
| *H3K9* | | 5’ TGCCAAAGGTGTAAAGCTC 3’ | 5’ ATCCTTGCTTCGATTAGCC 3’ |
